# Digital Technologies and Biomarkers for Locomotor Capacity Assessment in Older Adults: Systematic Review

**DOI:** 10.2196/83814

**Published:** 2026-04-14

**Authors:** Shuhan Zhou, Jundan Huang, Jia Yu, Xiaoyang Li, Chi Zhang, Yinan Zhao, Mingyue Hu, Hui Feng

**Affiliations:** 1 Xiangya School of Nursing, Central South University, 172 Tongzipo Road, Yuelu District, Changsha, 410013, China, 86 15173121969; 2 Oceanwide Health Management Institute, Central South University, Changsha, China; 3 Hunan Engineering Research Center for Intelligent Medical Care, Central South University, Changsha, China

**Keywords:** locomotor capacity, mobility, digital technology, digital biomarkers, assessment tools

## Abstract

**Background:**

Locomotor capacity, encompassing endurance, balance, muscle strength, muscle function, muscle power, and joint function of the body, is a key determinant of functional ability in older adults. Assessment tools based on digital technologies for objectively assessing locomotor capacity are increasingly being developed, but their reliability, validity, and clinical potential remain underexplored.

**Objective:**

This systematic review aims to evaluate the current state of digital technologies, assess their validity and reliability for assessing locomotor capacity, and facilitate their effective implementation in clinical settings.

**Methods:**

Systematic literature searches were performed in 6 electronic databases from inception to March 7, 2025. Citation lists from the included studies and gray literature from Google Scholar were additionally searched. Studies focusing on the reliability and validity of digital technologies for assessing locomotor capacity in general older adults were included. Standardized forms were used to extract information on study characteristics, participant demographics, digital technology details, and validity and reliability results. Methodological quality assessment and rating of measurement properties were conducted in accordance with the COSMIN (Consensus-Based Standards for the Selection of Health Measurement Instruments) guidelines.

**Results:**

A total of 14 studies were included, of which 13 assessed balance using inertial measurement units, smartphones, balance boards, and force plates, and 1 assessed muscle power using smartphones. Fifty-one digital biomarkers were identified, including 47 for balance and 4 for muscle power assessment. Test-retest reliability coefficients ranged from 0.016 to 0.97, and validity was context specific. Overall, 13 studies demonstrated sufficient test-retest reliability and validity, whereas 1 study was rated as insufficient for convergent validity. Methodological quality was rated as “doubtful” or “inadequate” in 11 studies.

**Conclusions:**

This review provides a comprehensive summary of digital technologies for assessing locomotor capacity in older adults and identifies 51 digital biomarkers with generally acceptable reliability and validity. Unlike previous studies that focused on specific sensor types or disease-specific populations, this review integrates evidence across technologies within general older populations, providing insights into the clinical application potential of digital biomarkers as well as the key translational barriers limiting their real-world implementation. Specifically, existing digital technologies show considerable promise for early detection of functional decline, longitudinal monitoring, and informing personalized interventions. However, their clinical applicability remains constrained by limited assessment of certain locomotor components and by methodological shortcomings across current studies. Future research should prioritize rigorous, high-quality investigations that expand evaluation to a broader range of locomotor components in real-world settings while developing age-friendly tools with enhanced clinical interpretability.

## Introduction

With an aging population, physical disability among older adults is becoming a pressing public health issue [1]. Locomotor capacity is a key factor for maintaining independence in daily activities [2]. The World Health Organization defines locomotor capacity as “a state (static or dynamic over time) of the musculoskeletal system that encompasses endurance, balance, muscle strength, muscle function, muscle power, and joint function of the body” [3]. In older adults, locomotion limitations increase the risk of adverse outcomes, such as frailty, falls, hospitalization, and death [4]. The prevalence of positive screenings for locomotion limitation varies from 2.8% to 52% depending on the setting [5].

Traditional methods for evaluating locomotor capacity, such as the Short Physical Performance Battery [6], exhibit several limitations, including the requirement for specialized assessor training, susceptibility to assessor bias, and ceiling effects [7,8]. Given these limitations, there is growing interest in developing assessment tools using digital technologies that offer more objective, quantifiable, and frequent monitoring of locomotor capacity [9-11]. Moreover, these frequent measures can capture short-term intraindividual fluctuations that may be early and sensitive indicators of negative trends and critical transitions [12]. For example, Kumar et al [13] used a tri-axial accelerometer worn by older adults over 48 hours to identify continuous walking bouts of 20, 30, 40, 50, and 60 seconds without pauses. They found that gait variability, asymmetry, and irregularity produced a sensitivity of 76.8% and specificity of 80% between robust and physical prefrail or frail individuals. By identifying these early signs from an individual’s typical mobility pattern, digital biomarkers have the potential to contribute to early risk prediction models for functional decline and disability, offering insights that conventional assessments may miss [14]. Digital technologies for measuring digital biomarkers can be categorized into 3 types [15]. First, wearable devices, such as wrist-worn accelerometers and portable sensors, provide continuous monitoring of movement patterns. Second, smartphones or applications are used for assessment. Third, nonvisual technologies enable passive and unobtrusive measurement in sensor-equipped environments.

Growing empirical evidence highlights the potential of digital technologies for assessing locomotor capacity. For instance, Burq et al [16] developed smartwatch-based unsupervised active tests of motor function in Parkinson disease and established the analytical validity of associated digital measures. Zhu et al [10] explored the use of inertial measurement units (IMUs) for assessing lower limb muscle strength during sit-to-stand transfers in older adults. Smartphone-based accelerometry data measured during sit-to-stand tests were also useful to identify subtle changes in locomotor capacity [17]. Several recent reviews [18-20] have summarized the application of digital technologies for assessing locomotor capacity. However, these reviews primarily focused on specific sensor types or disease populations, thereby overlooking other viable technologies and providing limited insight into assessments conducted in general older adult populations. In addition, the lack of robust evidence regarding the reliability and validity of digital assessment tools significantly limits their clinical utility [21]. The gap between research use and clinical application remains insufficiently established.

Therefore, this systematic review aimed to summarize digital technologies and biomarkers and evaluate their performance in assessing locomotor capacity in general older adults. The objectives were to (1) describe the types and mounting locations of digital technologies or devices used to measure overall or specific aspects of locomotor capacity; (2) identify digital biomarkers and corresponding assessment tasks derived from these technologies; and (3) assess the reliability and validity of digital technologies for detecting locomotor capacity.

## Methods

### Review Protocol and Amendments

This systematic review (registered in PROSPERO [International Prospective Register of Systematic Reviews]: CRD420251074143) followed the guidelines outlined in the PRISMA-COSMIN (Preferred Reporting Items for Systematic Reviews and Meta-Analyses-Consensus-Based Standards for the Selection of Health Measurement Instruments) for Outcome Measures Instruments in Systematic Reviews (OMIs) (Checklist 1) [22]. The only amendments to the registered review protocol were the inclusion of additional searches from Google Scholar and citation lists to ensure a more comprehensive review of the relevant literature.

### Search Strategies

The literature search was reported according to PRISMA-S to promote transparency of the search process [23]. The search terms were constructed using the PICO (Population, Intervention, Control, and Outcomes) framework, as displayed in Table S1 in Multimedia Appendix 1. Six databases, including PubMed (National Library of Medicine), Web of Science (Clarivate), Cochrane Library (Wiley), IEEE Xplore (IEEE), Scopus (Elsevier), and Embase (Elsevier), were systematically searched on March 7, 2025, without any restrictions or search filters. Major terms related to older adults, digital technology, assessment, and locomotor capacity were combined for the initial search, according to the review by Honvo et al [24]. The search strategy was developed in consultation with an experienced librarian and experts in geriatric medicine and digital health. In addition, we searched citation lists from included studies by browsing reference lists and gray literature on Google Scholar on November 17, 2025, limiting the search to the first 100 publications, as search results beyond this number rapidly lost relevance to the topic of the review [25]. No other online resources, such as conference proceedings or print journals, were specifically browsed for this review. No study registries were searched, and no additional studies were sought by contacting authors, manufacturers, or others. The search strategy is detailed in Table S2 in Multimedia Appendix 1. The search process was not updated after the initial search date.

### Eligibility Criteria

Following the PICO framework, the inclusion criteria were (1) study population (P): studies enrolling older adults as research subjects or separately reporting results on older people; (2) interventions (I): studies focusing on digital biomarkers (defined as objective, quantifiable physiological and behavioral measurements acquired through portable, wearable, implantable, or ingestible digital devices [15]), derived from digital technologies; (3) comparators (C): any or none; (4) outcomes (O): studies on the assessment of locomotor capacity (ie, endurance, balance, muscle strength, muscle function, muscle power, joint function) while examining the measurement properties of reliability and validity; and (5) studies written in English.

Reliability focused on 2 types [26,27]: (1) test‐retest reliability: the extent to which scores for patients who have not changed are the same for repeated measurements over time; and (2) measurement error: the systematic and random error of a patient’s score that is not attributed to true changes in the construct to be measured.

Validity prioritized 2 categories [27]: (1) criterion validity: the degree to which the scores of a device are an adequate reflection of a “gold standard”; and (2) hypothesis testing for construct validity: the degree to which the scores of a device are consistent with hypotheses, including convergent validity (measured by relationships to scores of other instruments) and discriminative validity (assessed through differences between relevant groups).

The exclusion criteria included the following: (1) systematic reviews and literature reviews; (2) books and other non–peer-reviewed literature; (3) studies conducted on populations with specific medical conditions (eg, Parkinson disease, stroke); (4) studies focusing on digital measures during rehabilitation exercises (eg, supervised therapeutic movements, postsurgical rehabilitation protocols, physical therapy interventions, and exercise programs designed for recovery or strength training); (5) studies lacking data on measurement properties; and (6) studies without full text.

### Study Selection

After the literature search, all identified studies were imported into EndNote 21, and duplicates were removed. Two authors independently screened the remaining titles and abstracts for potential eligibility and then retrieved the full-text articles of the selected studies. Any discrepancies regarding study selection were resolved through consultation with a third reviewer.

### Data Extraction

Two authors independently extracted data using standardized forms (Table S3 in Multimedia Appendix 1), and discrepancies were resolved through discussion among the 3 authors. Data items comprised: (1) characteristics of studies (first author, year of publication, and study design); (2) participant demographic information (sample size, age, sex distribution, and setting); (3) digital technology- or device-related information (type, number, assessment tasks, and digital biomarkers); and (4) results of validity and reliability.

### Quality Assessment

#### Overview

Quality assessment was independently undertaken by 2 authors based on the COSMIN guidelines [28], which comprised 3 substeps: the methodological quality of each study for a measurement property, the rated result of each study for a measurement property, and the pooled results of all available studies for a measurement property. Given the substantial heterogeneity in locomotion protocols and variables across the included studies, pooled results were not performed. In cases of disagreement during the evaluation process, a third researcher was consulted to reach a consensus.

#### Methodological Quality Assessment

For the methodological quality of the included studies, 4 measurement properties from the consensus-based COSMIN checklist [29] were evaluated: test-retest reliability, measurement error, criterion validity, and hypothesis testing for construct validity. As this checklist was originally developed to assess measurement properties of a patient-reported outcome measure, the extended version of COSMIN was used to replace the original boxes on test-retest reliability or measurement error [30]. The methodological quality of each study for a measurement property was categorized as very good, adequate, doubtful, or inadequate. The “worst score counts” principle was applied to determine the overall methodological quality of each measurement property. When a study reported a range of values or multiple values for a measurement property, the best value was selected for the measurement property rating [24].

#### Measurement Properties Quality Assessment

We first assessed each study for its measurement property quality according to the updated COSMIN criteria for good measurement properties [28]. Each property’s quality was rated as sufficient (+), insufficient (−), or indeterminate (?).

When at least 2 studies used the same protocols and variables, we synthesized the results. If the ratings were consistent across studies, the results from different studies for one measurement property were qualitatively summarized or pooled using meta-analysis, ultimately assigning a rating of “+” or “−.”

## Results

### Literature Search Result

**Figure 1.**
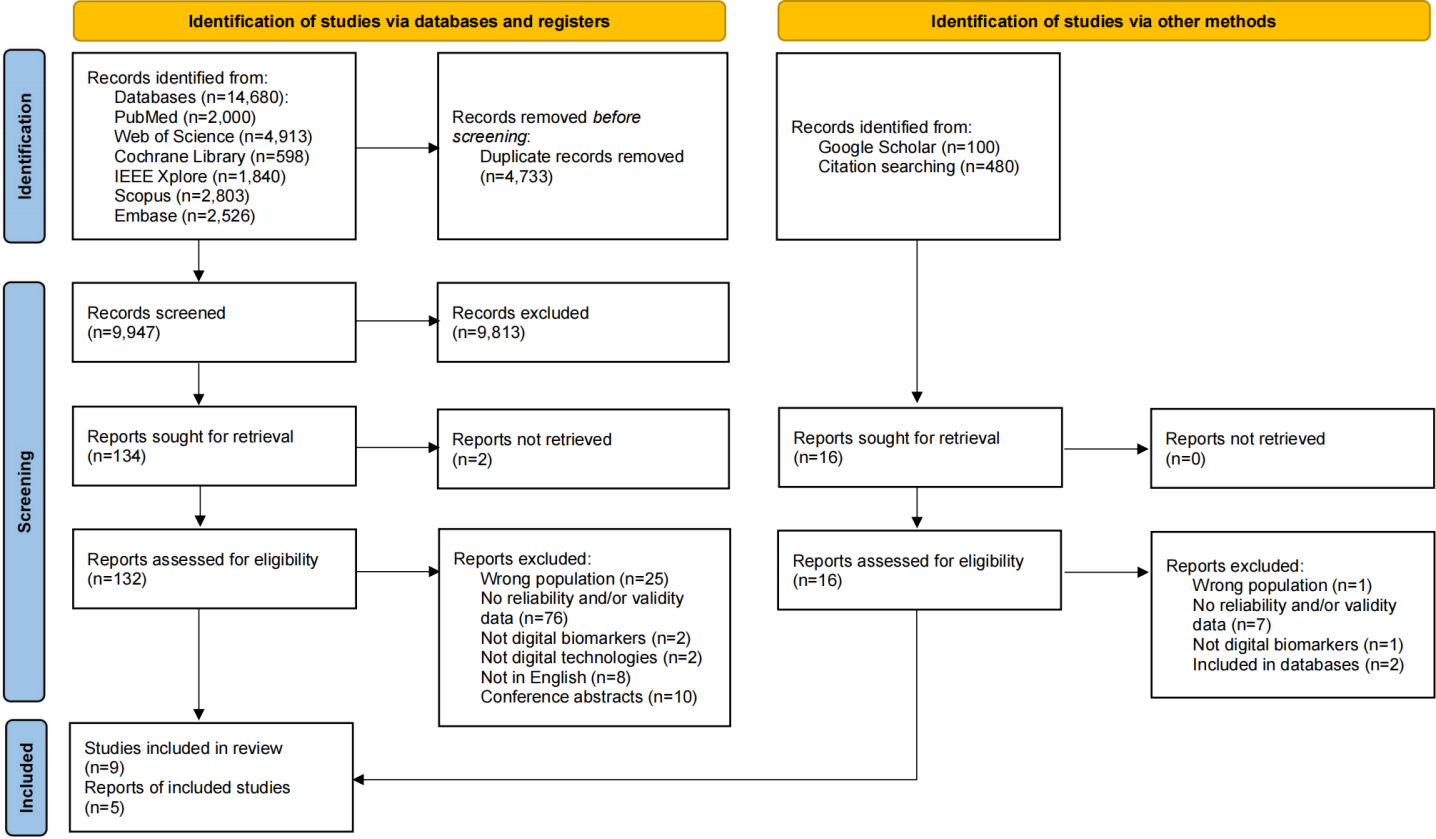
PRISMA (Preferred Reporting Items for Systematic Reviews and Meta-Analyses) flow diagram for the study selection process. This diagram illustrates the study selection process for the systematic review of digital measures of locomotor capacity in older adults, detailing each stage (from record identification through screening, eligibility assessment, and final inclusion) and providing explicit reasons for exclusion at each stage.

A total of 14,680 articles were identified from 6 databases, and 4,733 duplicates were removed. Following the initial screening of titles and abstracts, 123 articles were fully evaluated, with 9 of them eligible for inclusion in the review [31-39]. An additional 5 publications were obtained from reference lists [40,41] and gray literature [42-44]. The study selection process is presented in Figure 1.

### Study Characteristics

The main characteristics of the included studies and populations are summarized in Table 1. These studies were published between 2013 and 2024 and were conducted across 10 countries, primarily in the United States (n=3). Thirteen studies employed a cross-sectional design, while 1 adopted a pretest-posttest design. Across all studies, balance was the predominant assessment target (n=13), with only 1 study evaluating muscle power. No eligible studies assessed endurance, muscle strength, or joint function, and the exclusion reasons included incorrect population [45], lack of reliability and/or validity data [46], studies not involving digital biomarkers or technologies [47,48], and conference abstracts [49]. The sample sizes ranged from 12 to 248, with the majority from community-dwelling populations.

**Table 1.** Characteristics of the included studies.

Study	Country	Study design	Assessment target	Sample (count; age^a^ (y); male participants)	Population sources
Cerrito et al [31] (2015)	Switzerland	Cross-sectional study	Muscle power	16; 73.5 (10.4); 16	Retirement homes and community-dwelling
Chang et al [42] (2013)	China	Pretest-posttest design	Balance	20; 67.32 (3.43); NR^b^	NR
da Silva et al [32] (2013)	Brazil	Cross-sectional study	Balance	28; 69 (5); 8	Community-dwelling
De Groote et al [33] (2021)	Belgium	Cross-sectional study	Balance	97; 50‐90; 42	Community-dwelling
Greene et al [34] (2022)	Ireland	Cross-sectional study	Balance	168; 75.0 (7.2); 51	Community-dwelling
Harro and Garascia [40] (2019)	United States	Cross-sectional study	Balance	46; 67.7 (5.1); 22	Community-dwelling
Kuntapun et al [35] (2020)	Thailand	Cross-sectional study	Balance	12; 75.6 (5.6); 3	NR
Levy et al [36] (2018)	United States	Cross-sectional study	Balance	49; 71.3 (7.3); 23	Community-dwelling
McManus et al [37] (2022)	Ireland	Cross-sectional study	Balance	248; 74.9 (6.5); 91	Community-dwelling
Okada et al [38] (2024)	Japan	Cross-sectional study	Balance	27; 74.7 (7.1); 7	Community-dwelling
Olsen et al [43] (2023)	New Zealand	Cross-sectional study	Balance	34; 42‐94; 14	Community-dwelling
Pooranawatthanakul and Siriphorn [44] (2023)	Thailand	Cross-sectional study	Balance	20; 70.85 (4.09); 3	Community-dwelling
Scaglioni-Solano and Aragón-Vargas [41] (2013)	Costa Rica	Cross-sectional study	Balance	37; 69 (8); NR	Exercise groups
Zhou et al [39] (2021)	United States	Cross-sectional study	Balance	15; 71.4 (5.9); 7	Community-dwelling

a Age values are given as mean (SD) or range.

b NR: not reported.

### Balance Assessment

#### Device Type and Number

Table 2 provides an overview of the devices, tasks, variables, missing data, and data denoising methods. In terms of the assessment of balance, the most common device type was a smartphone (n=5), followed by a force plate (n=3), an IMU (n=2), a balance board (n=2), and an infrared depth sensor (n=1). All 13 studies used a single device for data collection. Two studies further assessed the reliability and validity across different sensor placement locations.

**Table 2.** Description of devices, tasks, variables, and data processing in the included studies.

Study	Devices (type; number)	Task settings	Device variables	Missing data and handling methods	Data denoising methods
Balance					
Chang et al [42] (2013)	Balance board; n=1	Three 10-s trials of stance with EO^a^, stance with EC^b^, and one-leg stance with EO	Center of gravity displacement	NR^c^	NR
da Silva et al [32] (2013)	Force plate; n=1	Three 30-s trials of the one-leg stance	COP^d^ area, COP sway RMS^e^ amplitude AP^f^, COP sway RMS amplitude ML^g^, mean velocity AP, mean velocity ML, mean frequency AP, and mean frequency ML	Complete data; NA^h^	Low-pass second-order Butterworth filter
De Groote et al [33] (2021)	Smartphone; n=1	Four 35-s trials of bipodal stance with EO, bipodal stance with EC, a dual task consisting of bipodal stance with EO and a cognitive task, and semi-tandem stance with EO	RMS acceleration ML, RMS acceleration AP, mean acceleration ML, and mean acceleration AP	Complete data; NA	Savitzky-Golay filter
Greene et al [34] (2022)	IMU^i^; n=1 (on thigh or sternum)	Five times sit-to-stand test	Balance model: including total time, coefficient of variation of sit-stand-sit time, mean time to stand up, spectral entropy angular velocity, mean Z-axis acceleration at stand start, and mean Z-axis acceleration at sit end	Complete data; NA	Butterworth IIR digital filter; signal validity checks
Harro and Garascia [40] (2019)	Force plate; n=1	Three standardized tests: limits of stability, motor control test, and sensory organization test	Limits of stability average end point excursion, motor control test average latency, sensory organization test composite equilibrium, and sensory organization test vestibular ratio scores	NR	NR
Kuntapun et al [35] (2020)	Smartphone; n=1 (attached horizontally at the level of the third lumbar vertebrae or rested on the right hip)	Walking 10 m at self-selected comfortable speed under three conditions: level walking, irregular surface walking, and obstacle crossing	COM^j^ AP displacement, COM ML displacement, and COM SI^k^ displacement	NR	Butterworth fourth-order low-pass filter
Levy et al [36] (2018)	Balance tracking system (consisting of a force plate and software); n=1	Six 20-s static standing trials of 3 with EO and 3 with EC	COP sway ML and COP sway AP	Complete data; NA	Dual-pass Butterworth filter
McManus et al [37] (2022)	IMU; n=1	Two 30-s trials of semi-tandem stance with EO and a narrow stance with EC	Balance score: including RMS acceleration magnitude, RMS acceleration ML, and RMS acceleration AP	Complete data; NA	Fourth order low-pass Butterworth filter
Okada et al [38] (2024)	Infrared depth sensor; n=1	20-s stepping-in-place test	Total movement distance, knee movement distance, maximum movement displacement, sway index, and step number	NR	NR
Olsen et al [43] (2023)	Smartphone; n=1	Four 30-s standing tasks: firm surface with EO, firm surface with EC, compliant surface with EO, and compliant surface with EC; and two 6-s walking tasks with EO, each performed four times: looking straight ahead and turning head	Postural stability, postural stability ML, postural stability AP, walking speed, mean step length, mean step time, step length variability, step time variability, step length asymmetry, and step time asymmetry	NR	NR
Pooranawatthanakul and Siriphorn [44] (2023)	Smartphone; n=1	Three standardized tests: the Modified Clinical Test of Sensory Interaction in Balance (ie, standing on a flat surface and a foam surface with EO or EC), a single-leg stance test, and a limit of stability test	RMS acceleration	Complete data; NA	Fourth-order Butterworth filter
Scaglioni-Solano and Aragón-Vargas [41] (2013)	Balance board; n=1	Four 30-s standing tasks (firm surface with EO, firm surface with EC, compliant surface with EO, and compliant surface with EC) and 10-s tandem stance test	COP displacement	Complete data; NA	Eighth-order Butterworth filter
Zhou et al [39] (2021)	Smartphone; n=1	Three 30-s trials of EO, EC, and dual task standing	Two-dimensional path length and RMS acceleration	Complete data; NA	Low-pass filter
Muscle power					
Cerrito et al [31] (2015)	Smartphone; n=1	Sit-to-stand movement	Total movement duration, peak force, rate of force development, and peak power	Complete data; NA	The signal was digitally low-pass filtered

a EO: eyes open.

b EC: eyes closed.

c NR: not reported.

d COP: center of pressure.

e RMS: root mean square.

f AP: anteroposterior.

g ML: mediolateral.

h NA: not applicable.

i IMU: inertial measurement unit.

j COM: center of mass.

k SI: superior-inferior.

#### Assessment Tasks

The included studies employed a diverse array of tasks designed to assess different aspects of balance. Nine studies [32,33,36,37,39,41-44] applied static standing tasks with eyes open and eyes closed, including bipodal, semi-tandem, narrow, dual task, and one-leg stance. Dynamic balance tasks were evaluated in 6 studies [34,35,38,40,43,44] using tasks such as walking, the 5-time sit-to-stand test, stepping-in-place test, limits of stability, motor control test, and sensory organization test. Two studies [43,44] conducted both static and dynamic standing tasks.

#### Device Variables

As presented in Table 3, a total of 47 distinct variables related to balance were identified from 13 studies, categorized as temporal (8, 17%), spatial (12, 25.5%), linear acceleration (11, 23.4%), angular velocity (1, 2.1%), position (11, 23.4%), and energy (4, 8.5%). Five studies [32,34,39,40,43] simultaneously assessed multiple categories of variables, primarily temporal, linear acceleration, and position variables.

**Table 3.** Classification and definitions of digital biomarkers of balance assessment.

Category and variable	Definition (unit)	Study
Temporal		
Walking speed	The mean of the ratios of step length to step time (m/s)	Olsen et al [43] (2023)
Mean step time	The mean of the time between two consecutive initial contacts of alternative feet (s)	Olsen et al [43] (2023)
Step time variability	The RMS^a^ of the SD of left step times and the SD of right step times (%)	Olsen et al [43] (2023)
Step time asymmetry	The percentage difference between left and right mean step times compared to the overall mean step time (%)	Olsen et al [43] (2023)
Total time to complete five times sit-to-stand test	The total time to complete five times sit-to-stand test (s)	Greene et al [34] (2022)
Coefficient of variation of sit-stand-sit time	The coefficient of variation of sit–stand–sit time across repetitions (%)	Greene et al [34] (2022)
Mean time to stand up	The mean time of the stand-up phase across repetitions (s)	Greene et al [34] (2022)
Motor control test average latency	The average time between the force plate translation and the individual’s active force responses in each leg (ms)	Harro and Garascia [40] (2019)
Spatial		
Mean step length	The mean of the AP^b^ distance between two consecutive initial contacts of alternative feet (m)	Olsen et al [43] (2023)
Step length variability	The RMS of the SD of left step lengths and the SD of right step lengths (%)	Olsen et al [43] (2023)
Step length asymmetry	The percentage difference between left and right mean step lengths compared to the overall mean step length (%)	Olsen et al [43] (2023)
Periodicity index	The step symmetry between the right and left step within a stride and the gait regularity across strides (%)	Olsen et al [43] (2023)
COM^c^ AP displacement	The average of minimum to maximum displacement in the AP direction across all step cycles (cm)	Kuntapun et al [35] (2020)
COM ML^d^ displacement	The average of minimum to maximum displacement in the ML direction across all step cycles (cm)	Kuntapun et al [35] (2020)
COM SI^e^ displacement	The average of minimum to maximum displacement in the SI direction across all step cycles (cm)	Kuntapun et al [35] (2020)
Total movement distance	The total linear distance determined from the sum total of step-by-step changes in head position from its initial to its final position (m)	Okada et al [38] (2024)
Knee movement distance	The mean of the sum of the three-dimensional movements of each knee joint point during stepping (m)	Okada et al [38] (2024)
Maximum movement displacement	The maximum displacement of the head along the movement path from its initial position (m)	Okada et al [38] (2024)
Sway index	The ratio of total movement distance to maximum movement displacement	Okada et al [38] (2024)
Step number in 20-s stepping-in-place test	The total number of foot contacts beginning with the return of the right foot to the ground after raising the right leg to initiate the test (steps)	Okada et al [38] (2024)
Linear acceleration		
RMS acceleration	RMS of acceleration in AP and ML directions (mm/s^2^)	Pooranawatthanakul and Siriphorn [44] (2023); Zhou et al [39] (2021)
RMS acceleration magnitude	RMS of the magnitude of the 3-axis acceleration	McManus et al [37] (2022)
RMS acceleration AP	RMS of COM acceleration in AP direction (m/s^2^)	De Groote et al [33] (2021); McManus et al [37] (2022)
RMS acceleration ML	RMS of COM acceleration in ML direction (m/s^2^)	De Groote et al [33] (2021); McManus et al [37] (2022)
Mean acceleration AP	Average absolute value of COM acceleration in AP directions (m/s^2^)	De Groote et al [33] (2021)
Mean acceleration ML	Average absolute value of COM acceleration in ML direction (m/s^2^)	De Groote et al [33] (2021)
Mean Z-axis acceleration at stand-start	The average Z-axis (vertical) accelerometer value recorded at the detected stand-start time across repetitions (m/s^2^)	Greene et al [34] (2022)
Mean Z-axis acceleration at sit-end	The average Z-axis (vertical) accelerometer value recorded at the detected sit-end time across repetitions (m/s^2^)	Greene et al [34] (2022)
Postural stability	The negative natural logarithm of the mean of the absolute acceleration along mediolateral, anterior–posterior, and vertical axes resultant vector (−ln[m/s^2^])	Olsen et al [43] (2023)
Postural stability ML	The negative natural logarithm of the absolute acceleration along ML axis resultant vector (−ln[m/s^2^])	Olsen et al [43] (2023)
Postural stability AP	The negative natural logarithm of the absolute acceleration along AP axis resultant vector (−ln[m/s^2^])	Olsen et al [43] (2023)
Angular velocity		
Spectral entropy angular velocity	The spectral entropy of the angular-velocity signal	Greene et al [34] (2022)
Position		
Center of gravity displacement	The average displacement of the center of gravity (cm)	Chang et al [42] (2013)
2D path length	The total length of the acceleration trajectories (mm)	Zhou et al [39] (2021)
COP^f^ displacement	The distance traveled by the COP (cm)	Scaglioni-Solano and Aragón-Vargas [41] (2013)
COP sway RMS amplitude AP	RMS amplitude of COP sway in AP direction (cm)	da Silva et al [32] (2013)
COP sway RMS amplitude ML	RMS amplitude of COP sway in ML direction (cm)	da Silva et al [32] (2013)
Mean velocity AP	Mean velocity of COP in AP direction (cm/s)	da Silva et al [32] (2013)
Mean velocity ML	Mean velocity of COP in ML direction (cm/s)	da Silva et al [32] (2013)
COP area	95% confidence ellipse area of COP (cm^2^)	da Silva et al [32] (2013)
Limits of stability average end point excursion	The average ratio of the initial distance traveled by the center of gravity on the primary attempt to reach each of the 8 multidirectional visual targets (%)	Harro and Garascia [40] (2019)
Sensory organization test composite equilibrium	The average equilibrium score (a measure of anterior/posterior sway during each trial compared to a theoretical stability limit of 12.5°) from conditions with fixed surface and normal vision, and fixed surface with eyes closed, added to the equilibrium scores from conditions with fixed surface and sway referenced vision, sway-referenced surface and normal vision, sway-referenced surface and EC, and sway-referenced surface and sway-referenced vision, then divided by the total number of trials	Harro and Garascia [40] (2019)
Sensory organization test vestibular ratio scores	The ratio of the equilibrium score in the sway-referenced surface, eyes closed condition to the fixed surface, normal vision condition	Harro and Garascia [40] (2019)
Energy		
Mean frequency AP	Mean frequency of COP in AP direction (Hz)	da Silva et al [32] (2013)
Mean frequency ML	Mean frequency of COP in ML direction (Hz)	da Silva et al [32] (2013)
COP sway AP	15.5 multiplied by the result of subtracting the sum of the force sensor data from the bottom left and bottom right corners from the sum of the force sensor data from the top left and top right corners and then dividing that result by the total sum of force sensor data from all four corners	Levy et al [36] (2018)
COP sway ML	24.25 multiplied by the result of subtracting the sum of the force sensor data from the top left and bottom left corners from the sum of the force sensor data from the top right and bottom right corners and then dividing that result by the total sum of force sensor data from all four corners	Levy et al [36] (2018)

a RMS: root mean square.

b AP: anteroposterior.

c COM: center of mass.

d ML: mediolateral.

e SI: superior-inferior.

f COP: center of pressure.

The device-derived variables extracted in the reviewed studies varied and were largely determined by the employed technology and the specific balance task. Five of the 7 studies utilizing smartphones or IMUs reported linear acceleration–based variables, with 4 studies [33,37,39,44] quantifying root mean square (RMS) acceleration. Among the 5 studies using force plates or balance boards, 4 reported position-based variables, primarily center of pressure (COP) metrics [32,40-42]. Additionally, 5 of the 6 studies involving dynamic tasks extracted temporal and spatial variables. Notably, 2 studies integrated multiple device-derived variables into composite balance scores [34,37].

#### Data Processing

Eight studies reported complete datasets and therefore did not require missing data handling, whereas 5 studies did not report the occurrence or management of missing data. Data denoising methods were described in 9 studies and primarily involved signal-filtering techniques, including low-pass Butterworth, Savitzky-Golay, and dual-pass filters.

#### Reliability

Table S4 in Multimedia Appendix 1 displays the findings on reliability and validity statistics. All studies investigated the test-retest reliability of balance assessment using the intraclass correlation coefficient (ICC). The reported ICC values varied considerably, ranging from 0.016 to 0.99, depending on the device types, tasks, or variable of choice. Chang et al [42] reported the highest test-retest reliability for the average displacement of gravity derived from a balance board during stance with eyes closed. Measurement error was reported in 6 studies, with standard error of measurement (SEM) values ranging from 0.02 to 16.1 and minimal detectable change (MDC) values ranging from 0.3 to 44.6.

When examined by device type and task setting, smartphones reported in 5 studies demonstrated ICCs from 0.016 to 0.97, with higher values in static tasks (0.50‐0.97) [33,39,43,44] and wider variability during dynamic tasks (0.016‐0.96) [35,43,44]. IMUs showed ICC values from 0.30 to 0.95, with more consistent reliability in static tasks (0.83‐0.95) [37] and lower, broader values in dynamic tasks (0.30‐0.91) [34]. Across both smartphones and IMUs, static tasks produced better and more consistent reliability than dynamic tasks. Force plates reported ICCs from 0.40 to 0.85 for static tasks [32,36] and 0.710 to 0.898 for dynamic tasks [40]. Balance boards were used exclusively for static assessments and yielded ICCs from 0.64 to 0.99 [41,42], whereas infrared depth sensors were applied only for dynamic assessments, with ICCs between 0.64 and 0.96 [38]. Regarding variable choice, temporal parameters showed ICCs from 0.30 to 0.91 [34,40,43], spatial parameters from 0.016 to 0.96 [35,38,43], linear acceleration metrics from 0.50 to 0.95 [33,34,37,39,43,44], angular velocity metrics from 0.67 to 0.81 [34], position-related metrics from 0.40 to 0.99 [32,39-42], and energy parameters from 0.72 to 0.83 [32,36]. As the reported SEM values corresponded to heterogeneous outcome variables with different units and scales, we did not perform comparisons across studies.

#### Validity

Of the 7 studies that investigated criterion validity, 4 used a force plate as the gold standard [33,36,39,41] and 3 used 3D motion capture [35,43,44]. In smartphone-based assessments compared with motion capture, spatial variables measured during dynamic tasks showed substantial variability, with correlations ranging from −0.51 to 0.98 [43]. In contrast, linear acceleration variables demonstrated consistently excellent correlations in static tasks, with coefficients of 0.91 to 0.98 [43,44], whereas dynamic tasks yielded a low, nonsignificant correlation of 0.37 [44]. When compared with force plates, smartphone-derived linear acceleration measures showed correlations of 0.14 to 0.82 in static tasks [33] and 0.38 to 0.60 in dynamic tasks [39]. Energy variables derived from force plates yielded Pearson correlations of 0.82 to 0.89 [36], and position variables from balance boards produced regression coefficients between 0.92 and 1.05 across static tasks [41].

Four studies [32,37,40,42] assessed convergent validity. Position variables derived from balance boards in static tasks showed significant positive correlations with smart balance master systems, ranging from 0.58 to 0.86 [42]. IMU-derived linear acceleration variables from static tasks had significant, fair correlations of 0.30 to 0.34 with Time Up and Go time [37]. In contrast, da Silva et al [32], using static tasks, and Harro et al [40], using dynamic tasks, reported generally low and nonsignificant correlations between position, energy, and temporal variables from force plates and clinical testing.

Discriminative validity was reported in 5 studies [32,34,37,38]. Two studies [32,42] compared young and older adults, and 2 [37,38] compared individuals with normal versus impaired balance, all demonstrating statistically significant between-group differences across multiple parameters, including position parameters from balance boards and force plates, and linear acceleration parameters from IMUs in static tasks, and spatial parameters from infrared depth sensors in dynamic tasks. One study [34] developed classification models using IMU data during dynamic tasks on the sternum and thigh, achieving 76.76% and 81.69% accuracy, respectively.

### Muscle Power Assessment

One study assessed muscle power using a smartphone during a sit-to-stand task [31]. The derived variables, including total movement duration, peak force, rate of force development, and peak power, were measured. Complete datasets were obtained, and the raw signals were filtered before analysis. Test-retest reliability of these variables ranged from 0.43 to 0.92. Measurement error, expressed as SEM, varied between 3.1 and 26.1. Convergent validity, evaluated by comparing smartphone measures with force plate data, showed a correlation coefficient ranging from 0.69 to 0.98, with peak power demonstrating a statistically significant correlation.

### Quality Assessment

#### Methodological Quality Assessment

The methodological quality and evaluation results of measurement properties are summarized in Table 4. For test-retest reliability, 9 of 14 studies reported “doubtful,” primarily because repeated assessments were conducted without familiar tests or adequate rest time, which may introduce variations and affect participant stability on the construct to be measured. For measurement error, 3 of 6 studies received a “doubtful” rating due to the absence of familiarization procedures, while the remaining studies were rated as very good.

**Table 4.** Methodological quality of studies and evaluation results of measurement properties based on COSMIN.

Study	Test-retest reliability		Measurement error		Criterion validity		Convergent validity		Discriminative validity	
	MQ^a^	ER^b^	MQ	ER	MQ	ER	MQ	ER	MQ	ER
Balance										
Chang et al [42] (2013)	D^c^	+^d^	NR^e^	NR	NR	NR	V^f^	+	D	+
da Silva et al [32] (2013)	V	+	V	?^g^	NR	NR	V	+	V	+
De Groote et al [33] (2021)	V	+	NR	NR	V	+	NR	NR	NR	NR
Greene et al [34] (2022)	D	+	NR	NR	NR	NR	NR	NR	D	+
Harro and Garascia [40] (2019)	V	+	V	?	NR	NR	I^h^	+	NR	NR
Kuntapun et al [35] (2020)	D	+	NR	NR	V	+	NR	NR	NR	NR
Levy et al [36] (2018)	D	+	D	?	V	+	NR	NR	NR	NR
McManus et al [37] (2022)	D	+	NR	NR	NR	NR	V	+	D	+
Okada et al [38] (2024)	D	+	NR	NR	NR	NR	NR	NR	V	+
Olsen et al [43] (2023)	D	+	D	?	V	+	NR	NR	NR	NR
Pooranawatthanakul and Siriphorn [44] (2023)	D	+	D	?	V	+	NR	NR	NR	NR
Scaglioni-Solano and Aragón-Vargas [41] (2013)	V	+	V	?	V	+	NR	NR	NR	NR
Zhou et al [39] (2021)	D	+	NR	NR	V	–^i^	NR	NR	NR	NR
Muscle power										
Cerrito et al [31] (2015)	V	+	V	?	NR	NR	I	+	NR	NR

a MQ: methodological quality.

b ER: evaluation results.

c D: doubtful.

d +: sufficient.

e NR: not reported.

f V: very good.

g ?: indeterminate.

h I: inadequate.

i −: insufficient.

Four studies evaluated criterion validity, and all were assessed as “very good.” Convergent validity was assessed in 5 studies, with 2 studies poorly describing the construct and measurement properties of the comparator instrument, resulting in an “inadequate” methodological quality rating. Of the 5 studies involving discriminative validity, 3 were rated “doubtful” owing to insufficient description of important subgroup characteristics.

#### Quality of Measurement Properties

As shown in Table 4, test-retest reliability, convergent validity, and discriminative validity were rated as sufficient (+) in 14, 5, and 5 studies, respectively. Measurement error was rated as indeterminate (?) in all 6 studies, as none defined a minimal important change (MIC). Criterion validity was rated as insufficient (−) in 1 study due to a correlation coefficient below 0.70 with the gold standard.

## Discussion

### Overview

This systematic review summarized digital technologies and biomarkers for assessing locomotor capacity in general older adults, including their validity and reliability. Fourteen studies were included, identifying 5 types of digital technologies and 51 digital biomarkers (47 for balance and 4 for muscle power). Twelve studies reported generally acceptable test-retest reliability and validity for balance, indicating promising potential. One study evaluated muscle power and demonstrated good reliability and validity, though further research is needed to validate these findings. However, caution is warranted when interpreting these results, as reliability and validity vary by context, and the overall strength of evidence remains limited. Future research should address other locomotor components and focus on developing assessments that simultaneously ensure suitability, reliability, and validity [50,51].

### Reliability

The test-retest reliability reported across included studies was acceptable, suggesting that digital technologies can produce stable measurements of locomotor capacity among older adults. Nevertheless, there was considerable variability in reported reliability coefficients, ranging from 0.016 to 0.97. This broad range highlights the nascent stage of the field and could be attributed to differences in device modalities, task settings, and the specific digital biomarkers extracted. Specifically, force plates generally exhibited more consistent test-retest reliability than smartphone- and IMU-based systems, which is commonly attributed to their direct measurement of ground reaction forces and COP trajectories and the absence of variability introduced by wearable sensor placement and soft-tissue artifacts [52,53]. Consistent with previous literature [54], portable sensors yielded higher and more consistent reliability for static tasks than for dynamic tasks [43,44], likely because dynamic conditions introduce increased movement variability, sensor micro-slippage, and more complex signal processing requirements. Variable choice also markedly influenced reliability. For example, in a wearable sensor-based sit-to-stand assessment, linear acceleration and angular velocity variables demonstrated higher test-retest reliability than temporal variables [34], likely because they are directly derived from continuous sensor signals, whereas temporal measures rely on event detection. Overall, these findings underscore the need for standardized sensor fixation and cautious use of integrated spatial metrics when employing mobile devices and depth sensors in locomotor assessment of older adults.

Only about half of the studies provided measurement error, such as SEM and MDC, which limited the assessment of whether observed differences reflect true change or fall within the bounds of random measurement error. Among those that reported measurement error, none defined a MIC, which prevents evaluation of whether the reported MDC values correspond to clinically meaningful change [55]. Therefore, the rating results of measurement error for relevant studies remained indeterminate. Future studies should systematically report SEM, MDC, and MIC to enable robust evaluation of measurement performance and to support meaningful clinical interpretation.

### Validity

Most studies showed acceptable validity, supporting the use of digital technologies to evaluate locomotor capacity. Regarding criterion validity, smartphone-derived linear acceleration measures showed strong correlations with 3D motion capture systems in static tasks but substantially weaker associations during dynamic conditions [44], highlighting the increased difficulty of accurately capturing complex movement patterns with smartphone sensors. In the studies comparing digital biomarkers with force plates, the correlation coefficients of linear acceleration variables derived from smartphones were generally lower than those of the energy variables from force plates and position variables from balance boards [33,36,41]. This could be attributed to inherent differences in the constructs measured; specifically, smartphones leveraged acceleration signals, whereas force plates and balance boards recorded ground reaction forces and COP.

As for convergent validity, the correlation between IMUs and Timed Up and Go tests was significant but low. This weak correlation may be because Timed Up and Go tests mainly assess rougher motor ability, whereas digital tools can measure balance dynamics and subtle changes more precisely [56]. Furthermore, the correlation between the force plate and clinical tests was generally low and nonsignificant in most cases. Clinical tests can measure functional balance abilities, whereas force plates can directly analyze balance related to reactive postural strategies and sensory integration [40,42].

The results of discriminative validity indicated that device-based measures were valid in differentiating balance performance between younger and older participants, which was consistent with a previous systematic review [57]. This not only reinforces the well-established physiological understanding of age-related declines in balance control, but more importantly, it positions digital biomarkers as sensitive tools for objectively quantifying these subtle yet significant changes associated with the aging process. The devices also distinguished between groups with impaired and normal balance. However, the criteria used to define balance groups varied across studies. This heterogeneity makes it difficult to compare the discriminative ability of different device-based measures.

### Digital Biomarkers

In this systematic review, we identified several digital biomarkers that demonstrated excellent reliability and validity for assessing locomotor capacity in older adults. Aligning with previous studies [58,59], COP parameters, including COP displacement, mean frequency anteroposterior (AP), COP sway AP, and COP sway mediolateral (ML), measured from balance boards and force plates during static standing tasks, exhibited strong reliability and validity for assessing balance. Furthermore, RMS acceleration, derived from smartphones, was also identified as a reliable and valid indicator of static balance. It has been frequently utilized in portable sensor-based balance assessments [33,37,39]. Beyond static balance measures, dynamic balance indicators such as walking speed, mean step time, and mean step length showed great reliability and validity for assessing dynamic balance. These gait-related biomarkers were also used to enhance the discrimination of gait disturbances and disease severity in individuals with Parkinson disease [56]. Additionally, total movement distance and step number in the 20-second stepping test measured by infrared depth sensors exhibited strong test-retest reliability and differentially discriminated between high- and low-balance subgroups. These 2 variables appeared to contribute to the differentiation of disordered balance and to suggest directions for future research.

Among studies focused on muscle power, Cerrito et al [31] demonstrated that smartphone-based peak power during sit-to-stand was a robust digital biomarker for evaluating lower-body muscle power in older adults. The use of peak power as a valid indicator of muscle power was further supported by studies employing linear transducers [60]. Furthermore, sit-to-stand muscle power was found to be a stronger predictor of falls, fractures, and physical independence than grip strength [61,62].

Integrating multiple variables can address multidimensional deficits that single measures miss [63] and has shown utility in frailty classification [64]. There is a growing trend toward constructing composite indices for balance impairment. Although 2 studies [34,37] integrating IMU-derived variables reported encouraging discriminative performance, these indices remain exploratory given the absence of rigorous validation and external replication. To establish their clinical applicability in balance assessment, future studies should focus on systematic performance evaluation and external validation.

### Quality of Evidence

Eleven studies were rated as having doubtful or inadequate methodological quality, leading to inflated or underestimated reliability and validity. Specifically, many studies lacked details about the familiarization process or the rest periods between repeated measurements. These omissions may introduce variation due to participant fatigue, learning effects, or other extraneous factors.

In addition, only 3 included studies reported a sample size greater than 50, and none used a sample size calculation. Small sample sizes may inflate the impact of individual variability, reduce statistical power, and result in unstable estimates of reliability and validity. In heterogeneous older adult populations, insufficient sample sizes further limit the generalizability of findings and increase uncertainty in measurement performance. Future research should prioritize adequately powered studies with predefined sample size considerations tailored to the intended measurement properties.

Only De Groote et al [33] met both good methodological quality and an adequate sample size, but the selected indicators, including RMS acceleration ML, RMS acceleration AP, mean acceleration ML, and mean acceleration AP, did not demonstrate satisfactory reliability and validity. Although many studies have reported good reliability and validity for other parameters, the methodological flaws in these studies necessitate caution when interpreting these findings.

### The Gap Between Research and Clinical Application

The digital transformation of healthcare in recent years has significantly advanced digital technologies, facilitating the transition of digital biomarkers from research contexts to clinical settings. Nevertheless, incorporating digital technology into healthcare to assess locomotor capacity in older adults comes with its own set of challenges.

One major challenge lies in balancing cost-effectiveness and measurement accuracy when selecting appropriate technologies to assess locomotor capacity. Force plates that measure postural sway by calculating COP are considered the gold standard for assessing balance [65]. However, their high cost and limited portability restrict their widespread clinical application [36]. More affordable and available alternatives, such as smartphones, IMUs, balance boards, and infrared depth sensors, have been suggested to demonstrate acceptable reliability and validity for assessing balance and locomotor performance [66]. In addition, emerging technologies are providing new perspectives for monitoring locomotor function. For example, Teel et al [67] explored the VR-based balance module for detecting persistent balance deficits in clinical concussion care, reporting a sensitivity of 85.7% and a specificity of 87.8%. As the field advances, hybrid technology integration may enable the capture of different aspects of locomotor function, thereby offering a more nuanced and holistic understanding of an individual’s physical health [61]. Manupibul et al [68] demonstrated that the integration of force-sensitive resistance sensors and IMUs provided complementary data, high accuracy, and enhanced performance to estimate the gait parameters.

The successful clinical translation of digital technologies for locomotor capacity requires careful consideration of user experience and usability, particularly ease of use among older adults with digital exclusion [69,70]. Tools or systems with complex interfaces or that require frequent manual adjustments may pose substantial barriers to adoption, compliance, and long-term use in older populations. Consistent findings from qualitative studies highlight ease of use as a key facilitator of technology adoption in this population [71,72]. Current research primarily involved participants performing specific, structured tasks under controlled conditions, resulting in potential missing data [33,39]. To achieve clinical effectiveness, future developments in locomotor capacity assessment tools should emphasize unobtrusive sensing, voice-based guidance, and minimal user interaction [73].

Context-specific performance also represents a key challenge for clinical application. Most of the studies included in this review collected data during the performance of predefined tasks under controlled conditions [35,40,44]. While certain digital biomarkers may demonstrate strong reliability and validity in specific tasks, their performance may vary across different tasks or settings. For example, RMS acceleration derived from smartphones in static tasks exhibited excellent criterion validity, but its performance deteriorated when applied to dynamic tasks [44]. Its performance can also be influenced by factors such as sensor placement, sampling frequency, and configured range [74]. Furthermore, the lack of standardized definitions and measurement protocols for these biomarkers complicates their reproducibility and generalization across studies.

To facilitate clinical application, interpretability represents an important consideration for future research. Interpretability can be considered from two perspectives: the clinical meaningfulness of digital biomarkers and the transparency of algorithms. The interpretability of digital biomarkers depends on how they reflect locomotor capacity, including the establishment of meaningful cut-off points that correspond to declines or improvements in mobility. Minimal clinically important difference is defined as the smallest change in a clinical outcome measure that is considered beneficial and meaningful to patients. By establishing minimal clinically important difference thresholds, clinicians can determine whether changes in digital biomarkers are clinically significant, thereby supporting decision-making [75]. With the integration of digital biomarkers, artificial intelligence-based algorithms may be employed, raising concerns about “black-box” systems, and clinical implementation should prioritize explainable artificial intelligence approaches to improve healthcare professionals’ and patients’ understanding of the measurement mechanism [76].

### Limitations

This systematic review offers a comprehensive overview of digital technologies for assessing locomotor capacity in older adults, providing integrated insights into digital health and physical function assessment. This systematic review has several limitations to note. First, we included only articles published in English, which may introduce publication bias. Second, although our search strategy was intentionally broad, the included studies predominantly focused on balance assessment, with other key components of locomotor capacity remaining underrepresented, reflecting a gap in the existing literature on other locomotor capacity components. Third, the included studies exhibited considerable heterogeneity, with variable study quality and assessment protocols. This variability is due to the emerging and expanding nature of this research field, which has yet to establish optimal assessment protocols and highlights a valuable strength of this review. Fourth, this systematic review predominantly focused on general older adults and excluded populations with specific diseases, such as Parkinson disease. The scope of our findings may be limited, and the generalizability to populations with specific diseases requires further investigation. Finally, this review may not have captured all emerging technologies, such as virtual reality and artificial intelligence, which are rapidly evolving and hold considerable potential for locomotor capacity assessment.

### Implications

Despite these limitations, the sufficient test-retest reliability and validity of the device-based assessment support its potential use in examining older adults’ balance and muscle power. Several recommendations need to be formulated. First, future studies should enhance methodological quality and adhere to COSMIN terminologies and recommendations [27,77]. Second, researchers should thoroughly evaluate both reliability and validity and compare the relevant measurement properties for various digital tools to facilitate the development of the optimal system. Third, alongside rigorous validation of measurement properties, future research should prioritize developing user-friendly digital tools. Current studies often employ task-specific protocols, limiting their widespread adoption in clinical practice and community settings. Fourth, variability in reliability and validity results indicated that no single device or method can be universally recommended for locomotor capacity assessment at this stage. Further studies need to standardize protocols and assess the robustness of these technologies in diverse real-world settings. Lastly, given the current diversity of technologies, the studies included in this review do not cover all existing tools, such as virtual reality and artificial intelligence applications. Future research is expected to explore the integration of these emerging technologies for a more precise and fine-grained assessment of locomotor capacity.

### Conclusion

This review offers an integrated understanding of the potential and limitations of digital technologies for assessing locomotor capacity in older adults, summarizing 51 digital biomarkers with generally acceptable reliability and validity. Unlike previous studies that predominantly targeted specific sensor types or disease-specific populations, our review incorporates diverse technologies and biomarkers across general older populations, offering a novel perspective on the potential for scalable, objective, and remote monitoring of changes in locomotor capacity related to aging. Our findings pave the way for developing digital biomarkers that enable the early detection of subtle declines in locomotor capacity, which is critical for timely intervention. However, the current evidence reveals several limitations, particularly task dependency and limited investigation of key locomotor components. The methodological quality of the included studies was generally doubtful, limiting the immediate clinical applicability of digital assessments. Future studies should expand the evaluation to a broader range of locomotor components and improve methodological quality with predefined sample size calculations. Furthermore, age-friendly assessments with enhanced clinical interpretability should be explored to facilitate clinical implementation. By addressing these challenges, digital biomarkers could become essential tools for enhancing the monitoring and management of older adults’ locomotor capacity, contributing to healthy aging strategies.

## Supplementary material

10.2196/83814 Multimedia Appendix 1 Search terms, search strategies, data extraction form, and description of reliability and validity in the included studies.

10.2196/83814 Checklist 1 PRISMA-COSMIN checklist.

## Abbreviations

AP: anteroposterior
COP: center of pressure
COSMIN: Consensus-Based Standards for the Selection of Health Measurement Instruments
ICC: intraclass correlation coefficient
IMU: inertial measurement unit
MDC: minimal detectable change
MIC: minimal important change
ML: mediolateral
OMI: Outcome Measures Instruments in Systematic Reviews
PICO: Population, Intervention, Control, and Outcomes
PRISMA: Preferred Reporting Items for Systematic Reviews and Meta-Analyses
PROSPERO: International Prospective Register of Systematic Reviews
RMS: root mean square
SEM: standard error of measurement
