# Supplementary material for: Digital Technologies and Biomarkers for Locomotor Capacity Assessment in Older Adults: Systematic Review
Source: J Med Internet Res. 2026 Apr 14;28:e83814. doi: 10.2196/83814 (PMC13078611; doi:10.2196/83814)
Supplement: Multimedia Appendix 1 [file jmir-v28-e83814-s001.docx]

**Supplementary Information**

**Table S1. Search terms developed using the PICO framework**

| PICO Item | Search terms |
| --- | --- |
| Population | “aged” OR “older adult*” OR “elder*” |
| Interventions | “digital health” OR “digital technology” OR “digital biomarker” OR “telehealth” OR “e-health” OR “sensor” OR “wearable” OR “smartphone” OR “smartwatch” |
| Comparators | Not applicable |
| Outcomes | “geriatric assessment” OR “assess*” OR “measur*” OR “estimat*” OR “evaluat*” OR “detect*” OR “monitor*”  AND  “physical endurance” OR “endurance” OR “postural balance” OR “balance” OR “posture equilibrium*” OR “postural equilibrium” OR “musculoskeletal equilibrium” OR “postural control*” OR “posture control*” OR “postural stability” OR “muscle strength” OR “muscle function” OR “muscle power” OR “joint function” OR “locomotor capacity” OR “locomotion” |

**Table S2. Search strategies**

| Database: PubMed  Search executed: Mar 7, 2025  Validated search filters used: None | | |
| --- | --- | --- |
| #1 | ("Aged"[Mesh] OR aged[Title/Abstract] OR "older adult*"[Title/Abstract] OR elder*[Title/Abstract]) | 4,318,427 |
| #2 | ("Digital Health"[Mesh] OR "Digital Technology"[Mesh] OR "Digital Health"[Title/Abstract] OR "Digital Technology"[Title/Abstract] OR "digital biomarker"[Title/Abstract] OR telehealth[Title/Abstract] OR e-health[Title/Abstract] OR sensor[Title/Abstract] OR wearable[Title/Abstract] OR smartphone[Title/Abstract] OR smartwatch[Title/Abstract]) | 251,468 |
| #3 | ("Geriatric Assessment"[Mesh] OR "Geriatric Assessment"[Title/Abstract] OR assess*[Title/Abstract] OR measur*[Title/Abstract] OR estimat*[Title/Abstract] OR evaluat*[Title/Abstract] OR detect*[Title/Abstract] OR monitor*[Title/Abstract]) | 13,290,035 |
| #4 | ("Physical Endurance"[Mesh] OR "Postural Balance"[Mesh] OR "Muscle Strength"[Mesh] OR "Locomotion"[Mesh] OR endurance[Title/Abstract] OR balance[Title/Abstract] OR "posture equilibrium*"[Title/Abstract] OR "postural equilibrium"[Title/Abstract] OR "musculoskeletal equilibrium"[Title/Abstract] OR "postural control*"[Title/Abstract] OR "posture control*"[Title/Abstract] OR "postural stability"[Title/Abstract] OR "muscle strength"[Title/Abstract] OR "muscle function"[Title/Abstract] OR "muscle power"[Title/Abstract] OR "joint function"[Title/Abstract] OR "locomotor capacity"[Title/Abstract] OR locomotion[Title/Abstract]) | 774,800 |
| #5 | #1 AND #2 AND #3 AND #4 | 2000 |
| Database: Web of Science  Search executed: Mar 7, 2025  Validated search filters used: None | | |
| #1 | TS=(aged OR "older adult*" OR elder*) | 12,238,407 |
| #2 | TS=("digital health" OR "digital technology" OR "digital biomarker" OR telehealth OR e-health OR sensor OR wearable OR smartphone OR smartwatch) | 5,284,463 |
| #3 | TS=("geriatric assessment" OR assess* OR measur* OR estimat* OR evaluat* OR detect* OR monitor*) | 39,914,563 |
| #4 | TS=(endurance OR balance OR "posture equilibrium*" OR "postural equilibrium" OR "musculoskeletal equilibrium" OR "postural control*" OR "posture control*" OR "postural stability" OR "muscle strength" OR "muscle function" OR "muscle power" OR "joint function" OR "locomotor capacity"OR "locomotion" ) | 2,595,778 |
| #5 | #1 AND #2 AND #3 AND #4 | 4,913 |
| Database: Cochrane Library  Search executed: Mar 7, 2025  Validated search filters used: None | | |
| #1 | MeSH descriptor: [Aged] in all MeSH produces | 276051 |
| #2 | (aged or older adult or older adults or elder or elderly):ti,ab,kw | 725569 |
| #3 | #1 or #2 | 725569 |
| #4 | MeSH descriptor: [Digital Health] explode all trees | 41 |
| #5 | MeSH descriptor: [Digital Technology] explode all trees | 24 |
| #6 | ("digital health" or "digital technology" or "digital biomarker" or telehealth or e-health or sensor or wearable or smartphone or smartwatch):ti,ab,kw | 22792 |
| #7 | #4 or #5 or #6 | 22792 |
| #8 | MeSH descriptor: [Geriatric Assessment] explode all trees | 2038 |
| #9 | ("geriatric assessment" or assess* or measur* or estimat* or evaluat* or detect* or monitor*):ti,ab,kw | 1422107 |
| #10 | #8 or #9 | 1422107 |
| #11 | MeSH descriptor: [Physical Endurance] explode all trees | 7606 |
| #12 | MeSH descriptor: [Postural Balance] explode all trees | 4415 |
| #13 | MeSH descriptor: [Muscle Strength] explode all trees | 9261 |
| #14 | MeSH descriptor: [Locomotion] explode all trees | 12061 |
| #15 | (endurance or balance or "posture equilibrium*" or "postural equilibrium" or "musculoskeletal equilibrium" or "postural control*" or "posture control*" or "postural stability" or "muscle strength" or "muscle function" or "muscle power" or "joint function" or "locomotor capacity"or "locomotion"):ti,ab,kw | 52972 |
| #16 | #11 or #12 or #13 or #14 or #15 | 70671 |
| #17 | #3 and #7 and #10 and #16 | 598 |
| Database: IEEE Xplore  Search executed: Mar 7, 2025  Validated search filters used: None | | |
| #1 | ("All Metadata":aged OR "All Metadata":"older adult*" OR "All Metadata":elder*) | 106,102 |
| #2 | ("All Metadata":"digital health" OR "All Metadata":"digital technology" OR "All Metadata":"digital biomarker" OR "All Metadata":telehealth OR "All Metadata":e-health OR "All Metadata":sensor OR "All Metadata":wearable OR "All Metadata":smartphone OR "All Metadata":smartwatch) | 818,214 |
| #3 | ("All Metadata":"geriatric assessment" OR "All Metadata":assess* OR "All Metadata":measur* OR "All Metadata":estimat* OR "All Metadata":evaluat* OR "All Metadata":detect* OR "All Metadata":monitor*) | 3,285,519 |
| #4 | ("All Metadata":endurance OR "All Metadata":balance OR "All Metadata":"posture equilibrium*" OR "All Metadata":"postural equilibrium" OR "All Metadata":"musculoskeletal equilibrium" OR "All Metadata":"postural control*" OR "All Metadata":"posture control*" OR "All Metadata":"postural stability" OR "All Metadata":"muscle strength" OR "All Metadata":"muscle function" OR "All Metadata":"muscle power" OR "All Metadata":"joint function" OR "All Metadata":"locomotor capacity"OR "locomotion") | 263,145 |
| #5 | ("All Metadata":aged OR "All Metadata":"older adult*" OR "All Metadata":elder*) AND ("All Metadata":"digital health" OR "All Metadata":"digital technology" OR "All Metadata":"digital biomarker" OR "All Metadata":telehealth OR "All Metadata":e-health OR "All Metadata":sensor OR "All Metadata":wearable OR "All Metadata":smartphone OR "All Metadata":smartwatch) AND ("All Metadata":"geriatric assessment" OR "All Metadata":assess* OR "All Metadata":measur* OR "All Metadata":estimat* OR "All Metadata":evaluat* OR "All Metadata":detect* OR "All Metadata":monitor*) AND ("All Metadata":endurance OR "All Metadata":balance OR "All Metadata":"posture equilibrium*" OR "All Metadata":"postural equilibrium" OR "All Metadata":"musculoskeletal equilibrium" OR "All Metadata":"postural control*" OR "All Metadata":"posture control*" OR "All Metadata":"postural stability" OR "All Metadata":"muscle strength" OR "All Metadata":"muscle function" OR "All Metadata":"muscle power" OR "All Metadata":"joint function" OR "All Metadata":"locomotor capacity"OR "locomotion") | 1840 |
| Database: Scopus  Search executed: Mar 7, 2025  Validated search filters used: None | | |
| #1 | TITLE-ABS-KEY ( aged OR "older adult*" OR elder* ) | 6,982,619 |
| #2 | TITLE-ABS-KEY ( "digital health" OR "digital technology" OR "digital biomarker" OR telehealth OR e-health OR sensor OR wearable OR smartphone OR smartwatch ) | 1,980,784 |
| #3 | TITLE-ABS-KEY ( "geriatric assessment" OR assess* OR measur* OR estimat* OR evaluat* OR detect* OR monitor* ) | 34,220,882 |
| #4 | TITLE-ABS-KEY ( endurance OR balance OR "posture equilibrium*" OR "postural equilibrium" OR "musculoskeletal equilibrium" OR "postural control*" OR "posture control*" OR "postural stability" OR "muscle strength" OR "muscle function" OR "muscle power" OR "joint function" OR "locomotor capacity" OR "locomotion" ) | 1,462,715 |
| #5 | #1 AND #2 AND #3 AND #4 | 2,803 |
| Database: Embase  Search executed: Mar 7, 2025  Validated search filters used: None | | |
| #1 | 'aged'/exp OR aged:ab,ti OR 'older adult*':ab,ti OR elder*:ab,ti | 5,073,556 |
| #2 | 'digital health'/exp OR 'digital technology'/exp OR 'digital health':ab,ti OR 'digital technology':ab,ti OR 'digital biomarker':ab,ti OR telehealth:ab,ti OR e-health:ab,ti OR sensor:ab,ti OR wearable:ab,ti OR smartphone:ab,ti OR smartwatch:ab,ti | 262,748 |
| #3 | 'geriatric assessment'/exp OR 'geriatric assessment':ab,ti OR assess*:ab,ti OR measur*:ab,ti OR estimat*:ab,ti OR evaluat*:ab,ti OR detect*:ab,ti OR monitor*:ab,ti | 17,540,026 |
| #4 | 'endurance'/exp OR 'body equilibrium'/exp OR 'muscle strength'/exp OR 'muscle function'/exp OR 'joint function'/exp OR 'locomotion'/exp OR endurance:ab,ti OR 'body equilibrium':ab,ti OR balance:ab,ti OR 'posture equilibrium*':ab,ti OR 'postural equilibrium':ab,ti OR 'musculoskeletal equilibrium':ab,ti OR 'postural control*':ab,ti OR 'posture control*':ab,ti OR 'postural stability':ab,ti OR 'muscle strength':ab,ti OR 'muscle function':ab,ti OR 'muscle power':ab,ti OR 'joint function':ab,ti OR 'locomotor capacity' OR 'locomotion':ab,ti | 861,118 |
| #5 | #1 AND #2 AND #3 AND #4 | 2,526 |

**Table S3. Data extraction form**

| Characteristics of the included studies | |
| --- | --- |
| Title |  |
| First author |  |
| Publication year |  |
| Country/Region |  |
| Study design |  |
| Assessment target (e.g. balance) |  |
| Characteristics of study population | |
| Sample size |  |
| Age |  |
| Male |  |
| Source (e.g. community-dwelling) |  |
| Digital technologies | |
| Type |  |
| Number |  |
| Location |  |
| Task |  |
| Digital biomarker |  |
| Missing data (proportion) |  |
| Handling methods |  |
| Data denoising methods |  |
| Results | |
| Reliability type and finding |  |
| Validity type and finding |  |

**Table S4. Description of reliability and validity in the included studies**

| Study | Reliability | | Validity | | |
| --- | --- | --- | --- | --- | --- |
|  | Test-retest reliability | Measurement error | Criterion validity | Hypothesis testing for construct validity | |
|  |  |  |  | Convergent validity | Discriminative validity |
| **Balance** |  |  |  |  |  |
| Chang (2013)[1] | ICC:  (stance with EO)  average displacement of the center of gravity 0.93 (0.86–0.97);  (stance with EC)  average displacement of the center of gravity 0.99 (0.97–0.99);  (one-leg stance with EO)  average displacement of the center of gravity 0.99 (0.98–0.99) | NR | NR | Against smart balance master system  Spearman correlation coefficients:  (stance with EO)  average displacement of the center of gravity 0.58 (p<0.05);  (stance with EC)  average displacement of the center of gravity 0.86 (p<0.05);  (one-leg stance with EO)  average displacement of the center of gravity 0.61 (p<0.05) | Young vs. older adults  (stance with EO)  average displacement of the center of gravity p≥0.05;  (stance with EC)  average displacement of the center of gravity p<0.05;  (one-leg stance with EO)  average displacement of the center of gravity p<0.05 |
| da Silva (2013)[2] | ICC:  COP area 0.60  COP sway RMS amplitude AP 0.51  COP sway RMS amplitude ML 0.40  mean velocity AP 0.85  mean velocity ML 0.82  mean frequency AP 0.82  mean frequency ML 0.72 | SEM:  COP area 6.66  COP sway RMS amplitude AP 1.98  COP sway RMS amplitude ML 1.82  mean velocity AP 0.90  mean velocity ML 1.3  mean frequency AP 0.09  mean frequency ML 0.08 | NR | Against one legged-stance time  Pearson’s correlation coefficients:  COP area -0.47 (p=0.002)  COP sway RMS amplitude AP -0.20 (p=0.315)  COP sway RMS amplitude ML -0.16 (p=0.419)  mean velocity AP -0.18 (p=0.353)  mean velocity ML -0.24 (p=0.235)  mean frequency AP -0.46 (p=0.013)  mean frequency ML -0.32 (p=0.094) | Young vs. older adults  COP area p=0.000  COP sway RMS amplitude AP p=0.006  COP sway RMS amplitude ML p=0.000  mean velocity AP p=0.000  mean velocity ML p=0.000  mean frequency AP p=0.000  mean frequency ML p=0.000 |
| De Groote (2021)[3] | ICC:  RMS acceleration ML, RMS acceleration AP, mean acceleration ML, and mean acceleration AP 0.5–0.9 | NR | Against force plate  Winsorized correlations:  RMS acceleration ML, RMS acceleration AP, mean acceleration ML, and mean acceleration AP 0.14–0.82 | NR | NR |
| Greene (2022)[4] | ICC:  (torso sensor)  total time 0.86 (0.82–0.90)  coefficient of variation of sit-stand-sit time 0.30 (0.10–0.47)  mean time to stand-up 0.83 (0.78–0.87)  spectral entropy angular velocity 0.67 (0.57–0.75)  mean Z-axis acceleration at stand-start 0.87 (0.83–0.90)  mean Z-axis acceleration at sit-end 0.85 (0.81–0.89);  (thigh sensor)  total time 0.86 (0.81–0.89)  coefficient of variation of sit-stand-sit time 0.57 (0.45–0.67)  mean time to stand-up 0.36 (0.17–0.51)  spectral entropy angular velocity 0.81 (0.75–0.85)  mean Z-axis acceleration at stand-start 0.91 (0.88–0.93)  mean Z-axis acceleration at sit-end 0.91 (0.89–0.93) | NR | NR | NR | Impaired vs. normal balance (based on Berg Balance Scale threshold of 53)  (Sternum sensor model)  accuracy 76.76%  sensitivity 46.34%  specificity 89.11%  PPV 63.33%  NPV 80.36%;  (thigh sensor model)  accuracy 81.69%  sensitivity 51.22%  specificity 94.06%  PPV 77.78%  NPV 82.61% |
| Harro (2019)[5] | ICC:  limits of stability average end point excursion 0.766 (0.575–0.872)  motor control test average latency 0.851 (0.730–0.917)  sensory organization test composite equilibrium 0.898 (0.816–0.944)  sensory organization test vestibular ratio scores 0.710 (0.476–0.839) | SEM:  limits of stability average end point excursion 9.0  motor control test average latency 4.4  sensory organization test composite equilibrium 3.14  sensory organization test vestibular ratio scores 0.1  MDC:  limits of stability average end point excursion 25.0  motor control test average latency 12.3  sensory organization test composite equilibrium 8.7  sensory organization test vestibular ratio scores 0.3 | NR | Pearson correlation coefficients  Against 10-m walk test free:  limits of stability average end point excursion 0.073 (p≥0.05)  motor control test average latency -0.328 (p<0.05)  sensory organization test composite equilibrium 0.127 (p≥0.05)  sensory organization test vestibular ratio scores 0.136 (p≥0.05);  Against 10-m walk test fast:  limits of stability average end point excursion 0.407 (p<0.05)  motor control test average latency -0.324 (p<0.05)  sensory organization test composite equilibrium 0.142 (p≥0.05)  sensory organization test vestibular ratio scores -0.029 (p≥0.05);  Against 6-minute walk test:  limits of stability average end point excursion 0.309 (p<0.05)  motor control test average latency -0.228 (p≥0.05)  sensory organization test composite equilibrium 0.123 (p≥0.05)  sensory organization test vestibular ratio scores -0.030 (p≥0.05);  Against Mini Balance Evaluation System Test:  limits of stability average end point excursion 0.347 (p<0.05)  motor control test average latency 0.148 (p≥0.05)  sensory organization test composite equilibrium 0.156 (p≥0.05)  sensory organization test vestibular ratio scores 0.204 (p≥0.05);  Against Functional Gait Assessment:  limits of stability average end point excursion 0.490 (p<0.05)  motor control test average latency 0.051 (p≥0.05)  sensory organization test composite equilibrium 0.245 (p≥0.05)  sensory organization test vestibular ratio scores 0.203 (p≥0.05) | NR |
| Kuntapun (2020)[6] | ICC:  (the third lumbar vertebrae and level walking)  COM AP displacement 0.902  COM ML displacement 0.757  COM SI displacement 0.860;  (the third lumbar vertebrae and irregular walking)  COM AP displacement 0.891  COM ML displacement 0.610  COM SI displacement 0.892;  (the third lumbar vertebrae and obstacle crossing)  COM AP displacement 0.544  COM ML displacement 0.701  COM SI displacement 0.582;  (right hip and level walking)  COM AP displacement 0.255  COM ML displacement 0.617  COM SI displacement 0.883;  (right hip and irregular walking)  COM AP displacement 0.252  COM ML displacement 0.745  COM SI displacement 0.719;  (right hip and obstacle crossing)  COM AP displacement 0.330  COM ML displacement 0.757  COM SI displacement 0.016 | NR | Against motion capture  Pearson’s correlation:  (the third lumbar vertebrae and level walking)  COM AP displacement -0.46  COM ML displacement -0.05  COM SI displacement 0.79;  (the third lumbar vertebrae and irregular walking)  COM AP displacement -0.51  COM ML displacement -0.19  COM SI displacement 0.65;  (the third lumbar vertebrae and obstacle crossing)  COM AP displacement 0.02  COM ML displacement 0.65  COM SI displacement 0.42;  (right hip and level walking)  COM AP displacement -0.15  COM ML displacement -0.28  COM SI displacement 0.20;  (right hip and irregular walking)  COM AP displacement -0.23  COM ML displacement -0.49  COM SI displacement 0.77;  (right hip and l obstacle crossing)  COM AP displacement -0.04  COM ML displacement -0.38  COM SI displacement 0.01 | NR | NR |
| Levy (2018)[7] | ICC:  (EO)  COP sway ML and COP sway AP 0.83 (0.71–0.89);  (EC)  COP sway ML and COP sway AP 0.83 (0.71–0.90) | SEM:  (EO)  COP sway ML and COP sway AP 3.47;  (EC)  COP sway ML and COP sway AP 7.0  MDC:  (EO)  COP sway ML and COP sway AP 9.6;  (EC)  COP sway ML and COP sway AP 19.4 | Against force plate  Pearson product moment coefficients r:  (EO)  COP sway ML and COP sway AP 0.82;  (EC)  COP sway ML and COP sway AP 0.89 | NR | NR |
| McManus (2022)[8] | ICC:  (EO)  RMS acceleration magnitude 0.85 (0.81–0.88)  RMS acceleration ML 0.92 (0.91–0.94)  RMS acceleration AP 0.83 (0.79–0.87);  (EC)  RMS acceleration magnitude 0.93 (0.92–0.95)  RMS acceleration ML 0.95 (0.93–0.96)  RMS acceleration AP 0.86 (0.83–0.89) | NR | NR | Against TUG time  Pearson moment correlations:  (EO)  balance score 0.30 (p<0.001);  (EC)  balance score 0.34 (p<0.001) | Impaired vs. normal balance (based on Berg Balance Scale threshold of 56)  (EO)  balance score p<0.001;  (EC)  balance score p<0.001 |
| Okada (2024)[9] | ICC:  total movement distance 0.96  knee movement distance 0.77  maximum movement displacement 0.64  step number 0.85 | NR | NR | NR | High vs. low-balance sub-group  (based on endpoint excursion)  total movement distance p≤0.05  knee movement distance p>0.05  maximum movement displacement p≤0.05  sway index p>0.05  step numbers p≤0.05;  (based on sway velocity)  total movement distance p≤0.05  knee movement distance p>0.05  maximum movement displacement p≤0.05  sway index p≤0.05  step numbers p>0.05 |
| Olsen (2023)[10] | ICC:  (task 1: firm EO)  postural stability 0.82 (0.7–0.9)  postural stability ML 0.88 (0.79–0.93)  postural stability AP 0.71 (0.54–0.84);  (task 2: firm EC)  postural stability 0.75 (0.61–0.85)  postural stability ML 0.79 (0.66–0.88)  postural stability AP 0.65 (0.48–0.79);  (task 3: compliant EO)  postural stability 0.78 (0.6–0.89)  postural stability ML 0.82 (0.62–0.89)  postural stability AP 0.76 (0.61–0.87);  (task 4: compliant EC)  postural stability 0.8 (0.6–0.9)  postural stability ML 0.78 (0.58–0.89)  postural stability AP 0.76 (0.59–0.89);  (task 5: walking looking straight ahead)  walking speed 0.82 (0.6–0.91)  periodicity index 0.74 (0.58–0.85)  mean step length 0.94 (0.84–0.97)  mean step time 0.76 (0.55–0.88)  step length variability 0.53 (0.33–0.7)  step time variability 0.38 (0.18–0.59)  step length asymmetry 0.75 (0.61–0.86)  step time asymmetry 0.64 (0.46–0.78);  (task 6: walking head turning)  walking speed 0.91 (0.83–0.95)  periodicity index 0.7 (0.54–0.82)  mean step length 0.96 (0.94–0.98)  mean step time 0.86 (0.72–0.93)  step length variability 0.45 (0.24–0.64)  step time variability 0.51 (0.31–0.69)  step length asymmetry 0.65 (0.47–0.79)  step time asymmetry 0.41 (0.19–0.61) | SEM:  (task 1)  postural stability 0.1  postural stability ML 0.1  postural stability AP 0.1;  (task 2)  postural stability 0.1  postural stability ML 0.2  postural stability AP 0.2;  (task 3)  postural stability 0.1  postural stability ML 0.1  postural stability AP 0.1;  (task 4)  postural stability 0.2  postural stability ML 0.2  postural stability AP 0.2;  (task 5)  walking speed 0.05  periodicity index 2  mean step length 0.01  mean step time 0.02  step length variability 1  step time variability 2  step length asymmetry 2  step time asymmetry 2;  (task 6)  walking speed 0.05  periodicity index 3  mean step length 0.02  mean step time 0.02  step length variability 2  step time variability 2  step length asymmetry 2  step time asymmetry 3 | Against 12-camera 3D motion capture  Pearson’s product-moment correlation:  (task 1–4)  postural stability 0.98 (0.98–0.99)  postural stability ML 0.96 (0.95–0.97)  postural stability AP 0.97 (0.96–0.97);  (task 5)  walking speed 0.79 (0.7–0.86)  mean step length 0.73 (0.61–0.81)  mean step time 0.97 (0.96–0.98)  step length variability 0.29 (0.09–0.47)  step time variability 0.49 (0.31–0.63)  step length asymmetry 0.14 (-0.06–0.34)  step time asymmetry 0.2 (-0.01–0.39);  (task 6)  walking speed 0.87 (0.8–0.91)  mean step length 0.8 (0.71–0.87)  mean step time 0.98 (0.97–0.99)  step length variability 0.28 (0.06–0.47)  step time variability 0.36 (0.16–0.54)  step length asymmetry 0.06 (-0.16–0.28)  step time asymmetry 0.21 (-0.01–0.41) | NR | NR |
| Pooranawatthanakul (2023)[11] | ICC:  (the Modified Clinical Test of Sensory Interaction in Balance)  RMS acceleration 0.87;  (a single-leg stance test)  RMS acceleration 0.80;  (a limit of stability test)  RMS acceleration 0.90 | SEM:  (the Modified Clinical Test of Sensory Interaction in Balance)  RMS acceleration 0.76;  (a single-leg stance test)  RMS acceleration 3.32;  (a limit of stability test)  RMS acceleration 0.39  MDC:  (the Modified Clinical Test of Sensory Interaction in Balance)  RMS acceleration 1.70;  (a single-leg stance test)  RMS acceleration 7.43;  (a limit of stability test)  RMS acceleration 0.86 | Against 3D motion system  Pearson’s correlation coefficients:  (the Modified Clinical Test of Sensory Interaction in Balance)  RMS acceleration 0.91 (p<0.001);  (a single-leg stance test)  RMS acceleration 0.91 (p<0.001);  (a limit of stability test)  RMS acceleration 0.37 (p=0.107) | NR | NR |
| Scaglioni-Solano (2013)[12] | ICC:  (firm EO)  CoP displacement 0.72 (0.54–0.84);  (firm EC)  CoP displacement 0.64 (0.39–0.78);  (compliant EO)  CoP displacement 0.85 (0.74–0.91);  (compliant EC)  CoP displacement 0.82 (0.70–0.89);  (tandem)  CoP displacement 0.82 (0.70–0.89) | SEM:  (firm EO)  CoP displacement 4.2  (firm EC)  CoP displacement 4.6  (compliant EO)  CoP displacement 5.1;  (compliant EC)  CoP displacement 16.1;  (tandem)  CoP displacement 5.8  MDC:  (firm EO)  CoP displacement 11.5;  (firm EC)  CoP displacement 12.7;  (compliant EO)  CoP displacement 14.1;  (compliant EC)  CoP displacement 44.6;  (tandem)  CoP displacement 16.1 | Against force plate  Regression coefficients:  (firm EO)  CoP displacement 0.96 (p<0.0005);  (firm EC)  CoP displacement 1.05 (p<0.0005);  (compliant EO)  CoP displacement 0.96 (p<0.0005);  (compliant EC)  CoP displacement 0.97 (p<0.0005);  (tandem)  CoP displacement 0.92 (p<0.0005) | NR | NR |
| Zhou (2021)[13] | ICC:  (EO)  2-D path length 0.95 (0.89–0.98)  RMS acceleration 0.94 (0.88–0.97);  (EC)  2-D path length 0.97 (0.94–0.99)  RMS acceleration 0.93 (0.87–0.97);  (dual-task)  2-D path length 0.87 (0.77–0.93)  RMS acceleration 0.8 (0.62–0.9) | NR | Against force plate  Spearman’s correlation:  2-D path length 0.60 (p<0.0001)  RMS acceleration 0.38 (p<0.0001) | NR | NR |
| **Muscle power** |  |  |  |  |  |
| Cerrito (2015)[14] | ICC:  total movement duration 0.92  peak force 0.88  rate of force development 0.43  peak power 0.91 | SEM:  total movement duration 6.9  peak force 3.1  rate of force development 19.7  peak power 26.1 | NR | Against force plate  Pearson’s correlation coefficients:  total movement duration 0.98 (p≥0.05)  peak force 0.86 (p≥0.05)  peak power 0.69 (p<0.05) | NR |

Abbreviations: ICC, intraclass correlation coefficient; EO, eyes open; EC, eyes closed; NR, not reported; COP, center of pressure; RMS, root mean square; AP, anteroposterior; ML, mediolateral; SEM, standard error of measurement; PPV, positive predictive value; NPV, negative predictive value; MDC, minimal detectable change; SI, superior-inferior; TUG, Timed Up and Go

**Reference**

1. Chang WD, Chang WY, Lee CL, Feng CY. Validity and reliability of wii fit balance board for the assessment of balance of healthy young adults and the elderly. J Phys Ther Sci. 2013 Oct;25(10):1251-3. PMID: 24259769. doi: 10.1589/jpts.25.1251.

2. da Silva RA, Bilodeau M, Parreira RB, Teixeira DC, Amorim CF. Age-related differences in time-limit performance and force platform-based balance measures during one-leg stance. Journal of Electromyography and Kinesiology. 2013 Jun;23(3):634-9. PMID: 23403137. doi: 10.1016/j.jelekin.2013.01.008.

3. De Groote F, Vandevyvere S, Vanhevel F, De Xivry J-JO. Validation of a smartphone embedded inertial measurement unit for measuring postural stability in older adults. Gait & Posture. 2021 Feb;84:17-23. PMID: 33260077. doi: 10.1016/j.gaitpost.2020.11.017.

4. Greene BR, Doheny EP, McManus K, Caulfield B. Estimating balance, cognitive function, and falls risk using wearable sensors and the sit-to-stand test. Wearable Technologies. 2022;3. PMID: 38486905. doi: 10.1017/wtc.2022.6.

5. Harro CC, Garascia C. Reliability and validity of computerized force platform measures of balance function in healthy older adults. J Geriatr Phys Ther. 2019 Jul/Sep;42(3):E57-e66. PMID: 29324510. doi: 10.1519/jpt.0000000000000175.

6. Kuntapun J, Silsupadol P, Kamnardsiri T, Lugade V. Smartphone monitoring of gait and balance during irregular surface walking and obstacle crossing. Frontiers in Sports and Active Living. 2020 Nov 27;2. PMID: 33345119. doi: 10.3389/fspor.2020.560577.

7. Levy SS, Thralls KJ, Kviatkovsky SA. Validity and reliability of a portable Balance Tracking System, BTrackS, in older adults. Journal of Geriatric Physical Therapy. 2018;41(2):102-7. PMID: 27893566. doi: 10.1519/JPT.0000000000000111.

8. McManus K, Greene BR, Ader LGM, Caulfield B. Development of data-driven metrics for balance impairment and fall risk assessment in older adults. IEEE Transactions on Biomedical Engineering. 2022;69(7):2324-32. PMID: 35025734. doi: 10.1109/TBME.2022.3142617.

9. Okada S, Takeshima N, Fujita E, Kohama T, Kusunoki M, Brechue WF. The stepping test, and infrared depth sensor, provide reliable measures of balance in community-dwelling older adults. Journal of physical therapy science. 2024 2024-Jan;36(1):9-20. PMID: 38186969. doi: 10.1589/jpts.36.9.

10. Olsen S, Rashid U, Allerby C, Brown E, Leyser M, McDonnell G, et al. Smartphone-based gait and balance accelerometry is sensitive to age and correlates with clinical and kinematic data. Gait Posture. 2023 Feb;100:57-64. PMID: 36481647. doi: 10.1016/j.gaitpost.2022.11.014.

11. Pooranawatthanakul K, Siriphorn A. Testing the validity and reliability of a new android application-based accelerometer balance assessment tool for community-dwelling older adults. Gait and Posture. 2023;104:103-8. PMID: 37379735. doi: 10.1016/j.gaitpost.2023.06.016.

12. Scaglioni-Solano P, Aragón-Vargas LF. Validity and reliability of the Nintendo Wii Balance Board to assess standing balance and sensory integration in highly functional older adults. Int J Rehabil Res. 2014 Jun;37(2):138-43. PMID: 24445863. doi: 10.1097/mrr.0000000000000046.

13. Zhou J, Jiang X, Yu W, Zhu H, Lo OY, Gouskova NA, et al. A smartphone app-based application enabling remote assessments of standing balance during the COVID-19 pandemic and beyond. IEEE Internet of Things Journal. 2021;8(21):15818-28. doi: 10.1109/JIOT.2021.3064442.

14. Cerrito A, Bichsel L, Radlinger L, Schmid S. Reliability and validity of a smartphone-based application for the quantification of the sit-to-stand movement in healthy seniors. Gait & Posture. 2015 Feb;41(2):409-13. PMID: 25467428. doi: 10.1016/j.gaitpost.2014.11.001.
